# Inhibitor of IκB kinase activity, BAY 11-7082, interferes with interferon regulatory factor 7 nuclear translocation and type I interferon production by plasmacytoid dendritic cells

**DOI:** 10.1186/ar3014

**Published:** 2010-05-14

**Authors:** Rie Miyamoto, Tomoki Ito, Shosaku Nomura, Ryuichi Amakawa, Hideki Amuro, Yuichi Katashiba, Makoto Ogata, Naoko Murakami, Keiko Shimamoto, Chihiro Yamazaki, Katsuaki Hoshino, Tsuneyasu Kaisho, Shirou Fukuhara

**Affiliations:** 1 First Department of Internal Medicine, Kansai Medical University, 10-15, Fumizono, Moriguchi, Osaka, 570-8506, Japan; 2 Laboratory for Host Defense, RIKEN Research Center for Allergy and Immunology, 1-7-22, Suehiro, Tsurumi-ku, Yokohama, Kanagawa, 230-0045, Japan; 3 Department of Allergy and Immunology, Osaka University Graduate School of Medicine, Osaka University, 2-2, Yamadaoka, Suita, Osaka, 565-0871, Japan; 4 Department of Supramolecular Biology, Graduate School of Nanobioscience, Yokohama City University, 1-7-29, Suehiro, Tsurumi-ku, Yokohama, Kanagawa, 230-0045, Japan

## Abstract

**Introduction:**

Plasmacytoid dendritic cells (pDCs) play not only a central role in the antiviral immune response in innate host defense, but also a pathogenic role in the development of the autoimmune process by their ability to produce robust amounts of type I interferons (IFNs), through sensing nucleic acids by toll-like receptor (TLR) 7 and 9. Thus, control of dysregulated pDC activation and type I IFN production provide an alternative treatment strategy for autoimmune diseases in which type I IFNs are elevated, such as systemic lupus erythematosus (SLE). Here we focused on IκB kinase inhibitor BAY 11-7082 (BAY11) and investigated its immunomodulatory effects in targeting the IFN response on pDCs.

**Methods:**

We isolated human blood pDCs by flow cytometry and examined the function of BAY11 on pDCs in response to TLR ligands, with regards to pDC activation, such as IFN-α production and nuclear translocation of interferon regulatory factor 7 (IRF7) *in vitro*. Additionally, we cultured healthy peripheral blood mononuclear cells (PBMCs) with serum from SLE patients in the presence or absence of BAY11, and then examined the inhibitory function of BAY11 on SLE serum-induced IFN-α production. We also examined its inhibitory effect *in vivo *using mice pretreated with BAY11 intraperitonealy, followed by intravenous injection of TLR7 ligand poly U.

**Results:**

Here we identified that BAY11 has the ability to inhibit nuclear translocation of IRF7 and IFN-α production in human pDCs. BAY11, although showing the ability to also interfere with tumor necrosis factor (TNF)-α production, more strongly inhibited IFN-α production than TNF-α production by pDCs, in response to TLR ligands. We also found that BAY11 inhibited both *in vitro *IFN-α production by human PBMCs induced by the SLE serum and the *in vivo *serum IFN-α level induced by injecting mice with poly U.

**Conclusions:**

These findings suggest that BAY11 has the therapeutic potential to attenuate the IFN environment by regulating pDC function and provide a novel foundation for the development of an effective immunotherapeutic strategy against autoimmune disorders such as SLE.

## Introduction

Although only a small fraction of cells, plasmacytoid dendritic cells (pDCs) represent a major source of type I interferons (IFNs) in peripheral blood mononuclear cells (PBMCs) and lymphoid tissues in both humans and mice [1,2], they play a central role in the innate antiviral immune response by their ability to rapidly produce robust amounts of type I IFNs upon viral infection. This function is through their selective expression of toll-like receptor (TLR)7 and TLR9, which respectively sense viral RNA and DNA within the early endosomes [3]. Recent studies have uncovered the molecular basis underlying the specialized ability of pDCs to mount their rapid and massive IFN response. The type I IFN production requires IFN regulatory factor (IRF)7 to be phosphorylated and translocated into the nucleus through rapid interaction with MyD88 and IRF7 [4]. pDCs are found to constitutively express high levels of IRF7 and the endogenous IRF7 facilitates a rapid type I IFN response that is independent of type I IFN receptor-mediated feedback signaling [3,5,6]. IRF7 is activated by forming cytoplasmic multiple signal-transducing complex with tumor necrosis factor (TNF) receptor-associated factor (TRAF)6 and interleukin (IL)-1 receptor-associated kinase (IRAK)4 through ubiquitylation and phosphorylation, and in turn interacts with TRAF3, IRAK1, osteopontin, and phosphatidylinositol-3 kinase (PI3K) [7-10]. A recent observation that pDCs barely express the translational inhibitors 4E-BP1 and 4E-BP2, which play a role in repression of Irf7 mRNA translation [11], could plausibly explain the constitutive expression of high levels of IRF-7 in pDCs. Thus, these unique molecular mechanisms endow pDCs with the specialized innate ability of IFN response upon viral infection.

Alternatively, a series of recent analyses have revealed that pDCs also play a pathogenic role in autoimmune diseases such as systemic lupus erythematosus (SLE) and psoriasis by their dysregulated production of type I IFNs through engagement of endosomal TLR9 by self-DNA with autoantibody [12-15]. Secretion of type I IFNs is believed to be a central molecular event that initiates and promotes the autoimmune process [12,14]. Type I IFNs induce the differentiation of myeloid DCs from monocytes, which in turn promote the differentiation of autoreactive CD4^+ ^T cells, CD8^+ ^T cells, and B cells. These autoreactive effectors injure tissues, resulting in the production of nucleic acid fragment and auto anti-nuclear antibody. This in turn induces the production of immune complexes containing self-DNA or RNA. The immune complexes further activate pDCs through TLRs in a sustained fashion, amplifying the vicious spiral based on the type I IFNs. Accordingly, pDCs and type I IFNs represent specific cellular and molecular targets in therapeutic strategies against these autoimmune diseases.

BAY11-7082 (BAY11), (E)-3-(4-methylphenylsulfonyl)-2-propenenitrile, was initially identified as a compound that inhibits the NF-κB pathway and leads to the decreased expression of endothelial cell adhesion molecules [16] and paw swelling in a rat adjuvant arthritis model [17]. Further studies searching for alternative therapeutic strategies against malignancies have shown that this compound is a potent inducer of apoptosis in a number of malignant cells such as in colorectal cancer [18] and breast cancer [19], as well as leukemia, myeloma cells, and lymphoma cells [20-24].

BAY11 is found to inhibit the upstream signaling process of NF-κB activation; namely it functions as an inhibitor for the action of the IκB kinase (IKK) complex, which consists of the catalytic kinase subunits IKKα and IKKβ [18,25].

Given a recent study showing that the activation of IRF7 depends on an IKK subfamily IKKα at the downstream of the TLR7/9-MyD88 pathway in pDCs [26], IKKα would be a potential molecular target for the treatment of type I IFN-related autoimmune diseases. As might be inferred from the function of BAY11 as inhibitor of IKK activity, we hypothesized that this compound could have the potential to repress the IFN response in pDCs through preventing IRF7 nuclear translocation, which may lead to an alternative treatment strategy for the autoimmune diseases.

We here show a novel function of BAY11, which inhibited IFN-α production by human pDCs as well as mouse pDCs upon TLR ligand activation by inhibiting the nuclear translocation of IRF7. We also showed its inhibitory effect *in vivo *by the observation that treatment with BAY11 attenuates the elevated level of serum type I IFNs in mice that were injected with TLR ligands. Our current results serve as the foundation for the development of an effective immunotherapeutic strategy to repress the autoimmune disorders induced by type I IFNs.

## Materials and methods

### Media and reagents

RPMI-1640 supplemented with 2 mM L-glutamine, 100 U/ml penicillin, 100 ng/ml streptomycin and heat-inactivated 10% fetal bovine serum (Biosource International, Camarillo, CA, USA) was used for cell cultures throughout the experiments. For human cell stimulation, we used 5 μM CpG-ODNs 2216 (Invivogen, San Diego, CA, USA), 100 μM Loxoribine (Invivogen), 1 μg/ml R848 (Invivogen), and 10 μg/ml Poly(I:C) (Invivogen). For mouse cell stimulation, we used 3 μg/ml polyuridine RNA (Poly U) (Sigma-Aldrich, St. Louis, MO, USA) in complex with lipofectamine 2000 (Invitrogen, Carlsbad, CA, USA) according to the manufactuer's protocol. BAY11-7082 (Alexis, San Diego, CA, USA) was dissolved in DMSO. DMSO was diluted in parallel to serve as a vehicle control.

### Cell isolation and culture

Human peripheral blood DC subsets (myeloid DCs and pDCs) were isolated from PBMCs from healthy adult donors, as described previously [3,27]. Written informed consent was obtained from all healthy adult donors. CD11c^+^/BDCA4^-^/lineage^-^/CD4^+ ^cells (as myeloid DCs) and CD11c^-^/BDCA4^+^/lineage^-^/CD4^+ ^cells (as pDCs) were sorted by FACS Aria^® ^(BD Biosciences, San Jose, CA, USA) to reach greater than 99% purity according to restaining with anti-BDCA1 or anti-BDCA2. Mouse splenic pDCs (CD11c^+^B220^+^CD11b^-^) were isolated by FACS Aria^® ^as described previously [28]. The DC subsets or PBMCs were preincubated for 15 minutes or 1 h with BAY11 (10^-9 ^to 10^-5 ^M) or vehicle. Poly(I:C), CpG, R848, Loxoribine, or poly U+lipofectamine was then added into this culture in flat-bottomed 96-well plates at 5 × 10^4 ^cells (2 × 10^5 ^cells for PBMCs) in the final 200 ml of medium per well for 24 h.

### Lupus PBMCs and serum, and preparation of necrotic cell supernatants

PBMCs and sera were obtained from three active SLE patients with low complements prior to steroid therapy and who satisfied five criteria in the American College of Rheumatology (ACR) classification for SLE [29]. Written informed consent was obtained for all SLE patients. All patients had anti-double-stranded DNA antibody. Necrotic cell supernatants were prepared from KM-H2 (human Hodgkin's Reed-Sternberg line), which was grown in RPMI with 20% of fetal bovine serum, and necrosis was induced by the *freeze-thaw *method. Briefly, freeze-thawing was performed in four cycles of both 10 minutes freezing at -80°C and thawing at 37°C. Lupus-PBMCs were stimulated with CpG-2216 with autologous 20% serum in flat-bottomed 48-well plates at 10^6 ^cells in 500 μl of medium per well. Alternatively, healthy PBMCs were stimulated with 20% lupus serum with or without 20% necrotic cell supernatant in flat-bottomed 96-well plates at 2 × 10^5 ^cells in 200 μl of medium per well. This study was approved by the Institutional Review Board of Kansai Medical University and the research was in compliance with the Helsinki declaration.

### *In vivo *assessment of cytokine productions

C57BL/6 mice (purchased from CLEA Japan, Meguro, Tokyo, Japan) were pretreated with BAY11 (10 mg/kg or 5 mg/kg bodyweight) or vehicle as control for 1 h intraperitonealy, followed by intravenous injection of poly U (50 μg/head) + *in vivo*-jetPEI (Polyplus-transfection, lllkirch, France) (according to the manufacturer's protocol). We analyzed the serum IFN-α levels at several time points (one, three, and six hours). All mice were maintained until used in the animal facilities under specific pathogen-free conditions. All animal researches were reviewed and approved by the Animal Ethical Committee of RIKEN Research Center.

### Analyses of cells

Human pDCs were stained with FITC-labeled CD86 (BD Biosciences) and then analyzed by FACScalibur^® ^(BD Biosciences). The production of cytokines in the culture supernatants after 24 hours was determined by ELISA (ELISA kits for human and mouse TNF-a and IL-12 p40 were purchased from R&D systems, (Minneapolis, MN, USA). ELISA Kits for human and mouse IFN-a were purchased from PBL Biomedical Laboratories (Piscataway, NJ, USA).

Intracellular cytokine staining in human pDCs was performed after eight hours of culture with different stimuli. Brefeldin A (10 μg/ml; Sigma-Aldrich, St. Louis, MO, USA) was added during the last two hours. After stimulation, cells were fixed and permeabilized using the FIX and PERM kit (Invitrogen, Carlsbad, CA, USA) and then stained with FITC-labeled anti-IFN-α2 mAb (Chromaprobe, Maryland Heights, MO, USA) phycoerythrin (PE)-labeled anti-TNF-α mAb (PBL Biomedical Laboratories), and allophycocyanin (APC)-labeled anti-BDCA4 mAb (Miltenyi Biotec, Bergisch Gladbach, Germany). Dead cells were excluded on the basis of side- and forward-scatter characteristics. In the viability assay, cells were washed with phosphate-buffered saline(PBS) containing 2 mM EDTA, and viable cells were counted in triplicate with trypan-blue exclusion of the dead cells. Viable cells were also evaluated using Propidium Iodide staining (Calbiochem, San Diego, CA, USA).

### Detection of p-NF-κB p65 expression

Human pDCs were stimulated with CpG-2216 or loxoribine at 90 minutes, and the cells were immediately fixed and stained with Alexa Fluor-488 anti-p-NF-κB p65 (pS529; BD Biosciences) according to BD Phosflow's instructions, and then analyzed by FACS calibur.

### Confocal microscopy

Cells were seeded on glass slides by cytospin and mounted, and were then fixed with 2% paraformaldehyde and permeabilized with 100% ice-cold methanol for 10 minutes at -20°C. Samples were labeled with rabbit polyclonal anti-human IRF-7 (H-246, Santa Cruz Biotechnology, Santa Cruz, CA, USA) and 4',6'-diamidino-2-phenylindole (DAPI). Anti-rabbit IgG-Cy5 (Invitrogen, Carlsbad, CA, USA) was used as secondary antibody. Images were acquired using a confocal microscope (LSM 510 META; Carl Zeiss, Inc. (Jena, Germany)).

## Results

### BAY11 inhibits IFN-α production from human PBMCs

**Figure 1:**
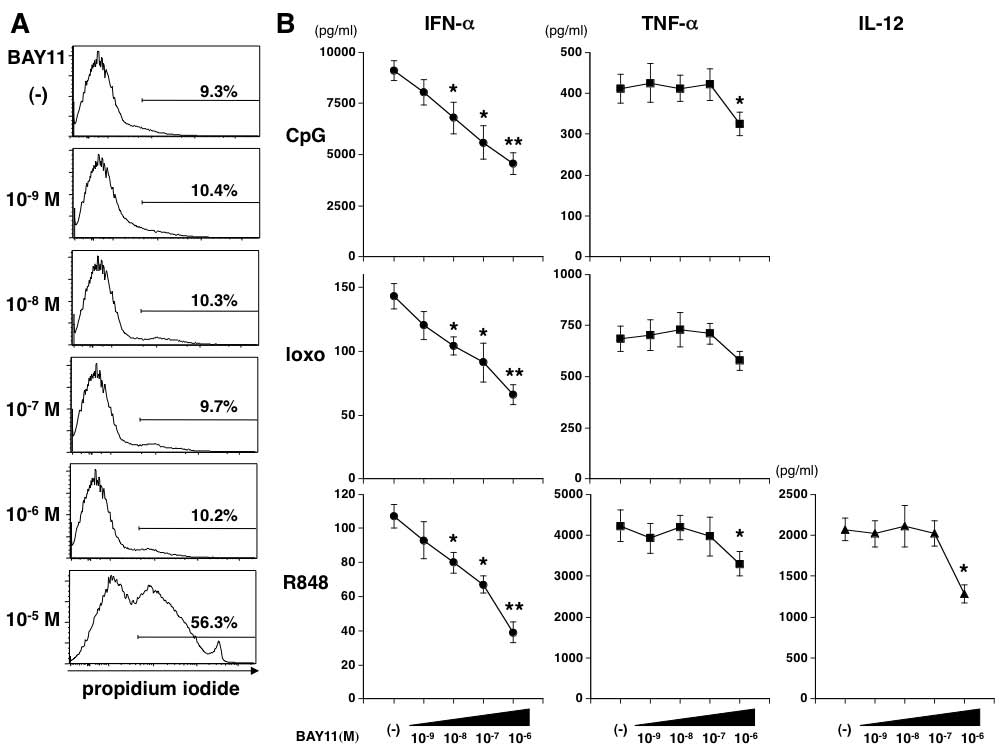
**BAY11 inhibits IFN-α production from human PBMCs**. **(A) **Human PBMCs were incubated for 24 h with BAY11 (10^-9 ^to 10^-5 ^M) or vehicle. Viable cells were analyzed by propidium iodide (PI) staining. Similar results were observed in five independent donors and the results of a representative experiment are shown. **(B) **Human PBMCs were preincubated for 15 minutes with BAY11 or vehicle, followed by addition of 5 μM CpG 2216, 1 μg/ml R848 and 100 μM loxoribine (loxo). After 24 h, the concentrations of IFN-α, TNF-α or IL-12 p40 in the culture supernatants were measured by ELISA. Data are shown as mean ± SEM of four independent donors. Statistical significance was determined using Mann-Whitney test (*P < 0.05 **P < 0.01).

In the first set of experiments, we assessed the immunomodulatory properties of BAY11 on human PBMCs. Because BAY11 was shown to have a cytotoxic activity at high concentrations [30], we analyzed the survival of PBMCs in the presence of different doses of BAY11 by propidium iodide (PI) staining (Figure 1A). Although a very high concentration (10^-5 ^M) of BAY11 induced cell death as shown by the more than 50% of PI expression in PBMCs, 10^-9 ^M to 10^-6 ^M of BAY11 did not increase PI-positive cells. We next measured the TLR ligand-induced cytokine production by PBMCs in the presence of 10^-9 ^M to 10^-6 ^M of BAY11. We found that IFN-α production by PBMCs in response to IFN-inducing TLR ligands (TLR9 ligand CpG 2216, TLR7/8 ligand R848, or TLR7 ligand loxoribine) were markedly inhibited in a dose-dependent manner (Figure 1B). By contrast, TNF-α production by PBMCs in response to these TLR ligands was only modestly inhibited by the 10^-6 ^M of BAY11. Similarly, IL-12 production induced by R848 was prevented by the 10^-6 ^M of BAY11.

### BAY11 directly inhibits IFN-α production from human pDCs

**Figure 2:**
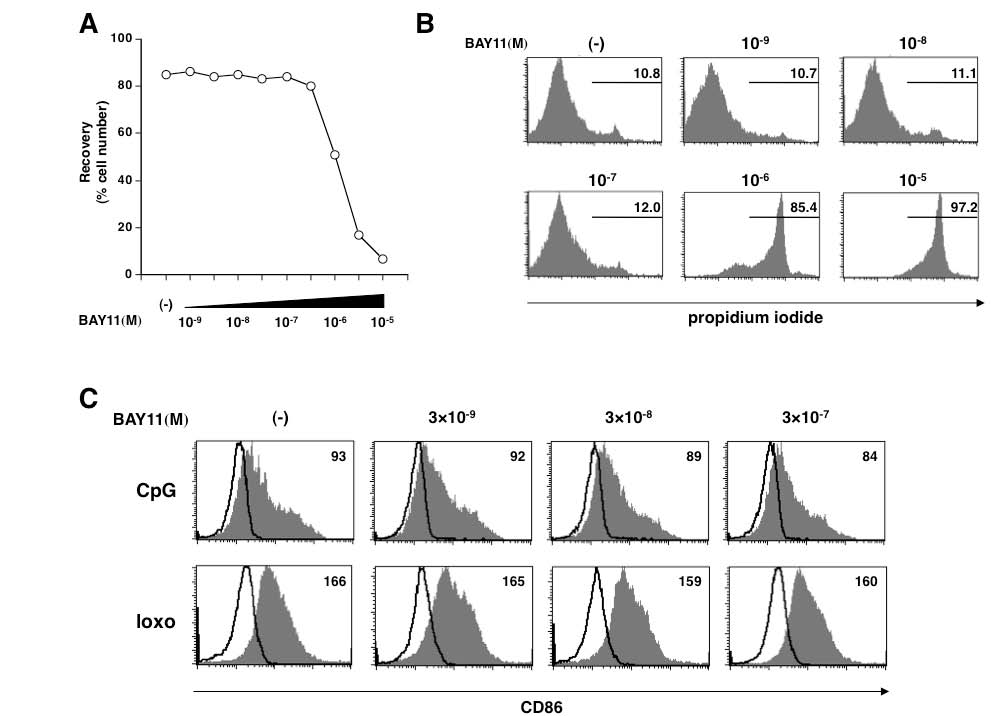
**Effects of BAY11 on pDC survival and maturation**. 10^-9 ^M to 3 × 10^-7 ^M of BAY11 does not affect viability or maturation of pDCs. Human pDCs were preincubated for 15 minutes with different concentrations of BAY11 (10^-9^, 3 × 10^-9^, 10^-8^, 3 × 10^-8^, 10^-7^, 3 × 10^-7^, 10^-6^, 3 × 10^-6 ^,10^-5 ^M) or vehicle. CpG 2216 or loxo were then added to the pDC cultures. After 24 h, viable cells were measured by a trypan-blue exclusion test **(A) **and PI staining **(B)**, and CD86 expression on pDCs was analyzed by flow cytometry **(C)**. Percentages of PI-positive cells are indicated in B. Numbers in the histograms (C) indicate the mean fluorescence intensity (MFI), which is calculated by the subtraction of MFI with the isotype control from that with CD86 mAb. Similar results were observed in three independent donors and the results of a representative experiment are shown.

Next, to investigate whether BAY11 functions directly on pDCs, as the major source of type I IFNs, to inhibit the IFN response, we used purified pDCs in the cultures with different doses of BAY11. Because pDCs are very fragile [27], we used a titration assay of BAY11 (10^-9 ^M to 10^-5 ^M) to test the viability of pDCs and determine the concentration range of BAY11 that does not induce cell death. Analysis of trypan-blue exclusion of the dead cells (Figure 2A) and PI staining (Figure 2B) showed that over 10^-6 ^M of BAY11 killed pDCs even in response to TLR-stimuli but that there were no significant differences in the rate of viable cells between the condition without BAY11 and with up to 3 × 10^-7 ^M of BAY11. Therefore, we thereafter used 10^-9 ^M to 3 × 10^-7 ^M of BAY11 for the following assays.

We further investigated the effect of BAY11 on the pDC maturation. Up to 3 × 10^-7 ^M of BAY11 did not influence the CD86 expression on pDCs in response to CpG or loxoribine (Figure 2C).

**Figure 3:**
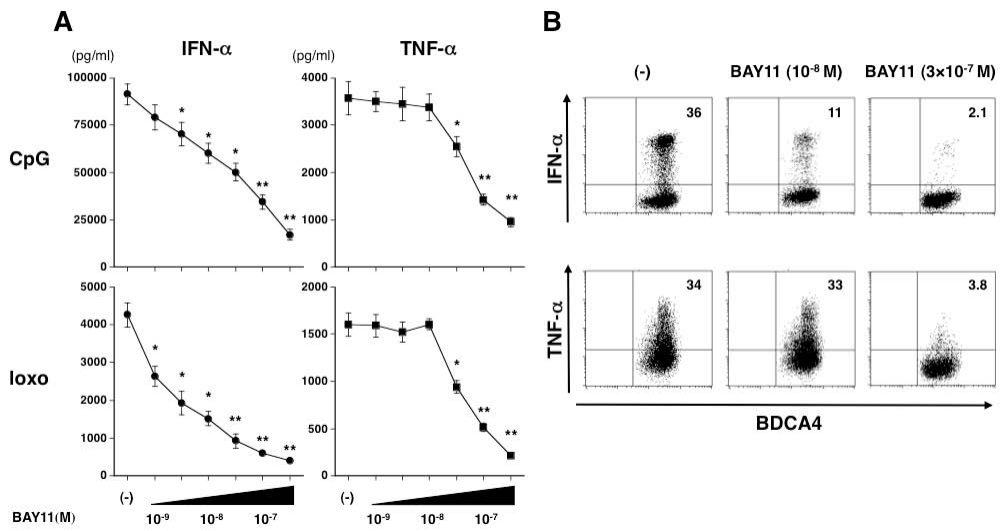
**BAY11 inhibits IFN-α production from human pDCs**. Human pDCs were preincubated for 15 minutes with different concentrations of BAY11 (10^-9^, 3 × 10^-9^, 10^-8^, 3 × 10^-8^, 10^-7^, 3 × 10^-7^) or vehicle. CpG 2216 or loxo were then added to the pDC cultures. **(A) **After 24 hours, the concentrations of IFN-α and TNF-α in the culture supernatants were measured by ELISA. Data are shown as mean ± SEM of four independent donors. Statistical significance was determined using Mann-Whitney test (*P < 0.05, **P < 0.01). **(B) **After eight hours of stimulation with CpG 2216, intracellular cytokine (IFN-α and TNF-α) expression and surface BDCA4 expression by pDCs were analyzed by flow cytometry. Percentages of the cytokine-producing pDCs are indicated in each dot-blot profile. Similar results were observed in three independent donors and the results of a representative experiment are shown.

We measured TLR-mediated cytokine production by purified pDCs in the presence or absence of BAY11. We found that IFN-α production by pDCs in response to CpG 2216 and loxoribine were severely impaired by BAY11 in a dose-dependent manner between 10^-9 ^M to 3 × 10^-7 ^M (Figure 3A). However, the inhibitory response of TNF-α production was more modest than that of IFN-α production. Namely, the TNF-α production was not prevented by a BAY11 concentration of between 10^-9 ^and 10^-8 ^M, which significantly inhibited the IFN-α production (Figure 3A). Further analysis with intracellular cytokine staining also showed severe defects in both IFN-α and TNF-α expression in CpG-stimulated pDCs after exposure to 3 × 10^-7 ^M of BAY11, but only a decrease in IFN-α- expressing cells after exposure to 10^-8 ^M (Figure 3B). These findings suggest that the effective dose of BAY11 on pDCs can be divided into three concentration ranges; low: 10^-9 ^M to 10^-8 ^M of BAY11, which selectively interfered with IFN-α production; medium: 10^-8 ^M to 3 × 10^-7 ^M of BAY11, which exhibited an inhibitory effect on the production of both IFN-α and TNF-α; and high: over 10^-6 ^M of BAY11, which had a cytotoxic impact.

### BAY11 is incapable of interfering with poly IC-induced IFN-α production from myeloid DCs

**Figure 4:**
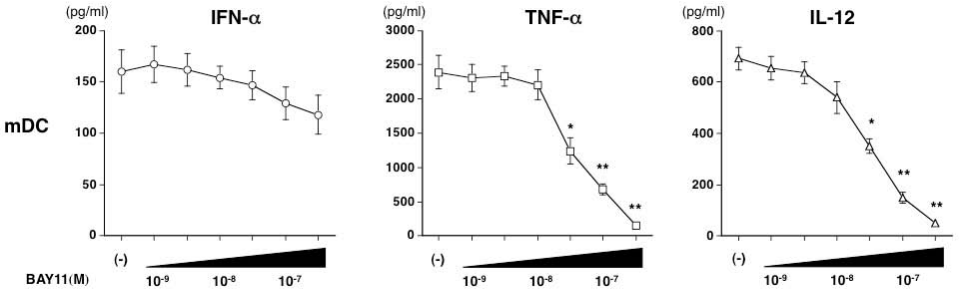
**BAY11 does not affect poly IC-induced IFN-α production**. Human myeloid DCs were pretreated for 15 minutes with different concentrations of BAY11 (10^-9^, 3 × 10^-9^, 10^-8^, 3 × 10^-8^, 10^-7^, 3 × 10^-7^) or vehicle, followed by addition of 10 μg/ml poly IC. After 24 hours, the concentrations of IFN-α, TNF-α and IL-12 in the culture supernatants were measured by ELISA. Data are shown as mean ± SEM of four independent donors. Statistical significance was determined using Mann-Whitney test (*P < 0.05, **P < 0.01).

In PBMCs, IFN-α production through TLR signaling mainly depends on pDCs. However, PBMCs contain monocytes and myeloid DCs, which can produce type I IFNs upon RNA recognition, though IFN-α production is much less than with pDCs [31]. Poly IC stimulated myeloid DCs to produce IL-12 and IFN-α through triggering endosomal TLR3 and cytosolic MDA5 [32]. We therefore examined whether BAY11 inhibits the production of these cytokines by myeloid DCs. Although TNF-α and IL-12 production were impaired by 10^-7 ^M of BAY11, IFN-α production was not significantly inhibited by doses of up to 3 × 10^-7 ^M of BAY11 (Figure 4). Up to 3 × 10^-7 ^M of BAY11 did not induce a PI-positive cell rate of myeloid DCs (data not shown).

### BAY11 inhibits nuclear translocation of IRF7 in pDCs

**Figure 5:**
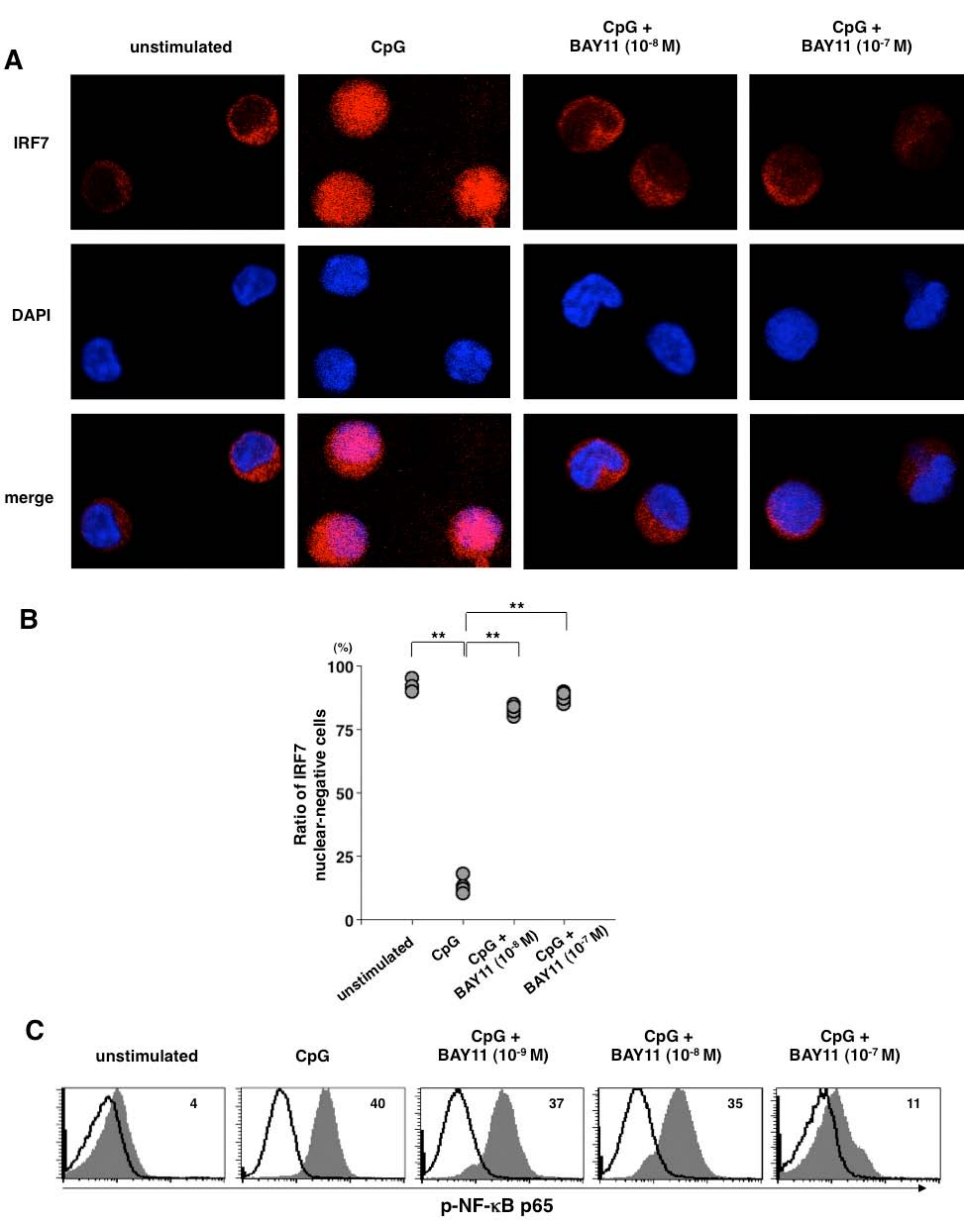
**BAY11 inhibits nuclear translocation of IRF7 and NF-κB phosphorylation in pDCs upon TLR-mediated activation**. Human pDCs were pretreated for 15 minutes with BAY11 (10^-9 ^to 10^-7 ^M) or vehicle, followed by addition of CpG 2216 and cultured for three hours. **(A, B) **Freshly isolated or activated pDCs were visualized by immunofluorescence with IRF7 antibody (Cy5, red) and nuclei staining with DAPI (blue). Similar results were observed in four independent donors and the representative cells are shown in (A). Cells without nuclear IRF7 expression were counted on the slide from four independent donors (B). Cells were regarded as negative when the expression level of IRF7 in the cytoplasm was higher and distinguishable from that in the nucleus, shown by DAPI staining. Ratio of nuclear IRF7-negative cells was analyzed by 50 cells in each donor. Statistical significance was determined using Mann-Whitney test (**P < 0.01). **(C) **Staining with anti-p-NF-κB p65 mAb (shaded) and isotype-matched control (solid line) for freshly isolated pDCs or activated pDCs are shown. Similar results were observed in three independent donors and the results of a representative experiment are shown.

Because the key molecular step in the type I IFN production by pDCs in response to ligand for TLR7 or TLR9 has been elucidated to be nuclear translocation of the constitutive expression of IRF7 [3,33], we assessed whether BAY11 inhibits this process in pDCs. Analysis with immunofluorescence microscopy revealed that IRF7 was constitutively expressed and localized in the cytoplasmic area of unstimulated pDCs (Figure 5A). After three hours stimulation of CpG, IRF7 was detected in the nucleus, as shown by colocalization with DAPI nuclear staining, indicating the nuclear translocation of IRF7. This colocalization of IRF7 and DAPI staining was prevented by the presence of 10^-8 ^M and 10^-7 ^M of BAY11 (Figure 5A). Thus, BAY11 helped retain IRF7 in the cytoplasm, indicating an inhibitory effect of IRF7 nuclear translocation in pDCs. To show this finding quantitatively, we counted the cell numbers with or without nuclear IRF7 expression in pDCs on the slide, as described [10]. The frequency of cells without IRF7 nuclear translocation was significantly augmented by BAY11 in response to CpG (Figure 5B). Thus, our result identifies that BAY11 acts as an inhibitor of IRF7 nuclear translocation and indicates that the inhibition of type I IFN production by BAY11 is due to its inhibitory function on the nuclear translocation of IRF7.

Unlike type I IFN production, inflammatory cytokine and chemokine production have been shown to be mostly through NF-κB activation [34]. Because BAY11 was initially identified as a potent inhibitor of NF-κB pathway, we confirmed its function in regard to NF-κB activation in pDCs. Analysis with flow cytometry (Figure 5C) showed that although 10^-9 ^M and 10^-8 ^M of BAY11 only slightly decreased the intensity of TLR-induced NF-κB phosphorylation, 10^-7 ^M of BAY11 strongly interfered with the NF-κB phosphorylation in accord with TNF-α production (Figure 3A).

### BAY11 inhibits both IFN-α production by lupus-PBMCs and lupus serum-induced IFN-α production

**Figure 6:**
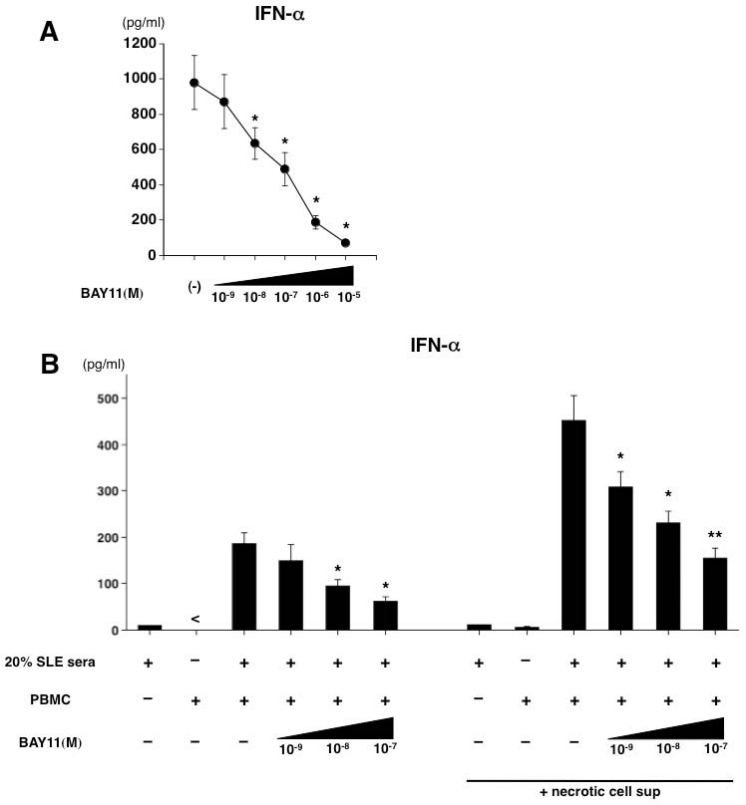
**BAY11 inhibits *in vitro *pathogenic conditioned IFN-α production**. **(A) **PBMCs from three patients with SLE were isolated and 1 × 10^6 ^cells were preincubated for 15 minutes with BAY11 (10^-9 ^to 10^-5 ^M) or vehicle in the presence of the autologous serum (at a final concentration of 20% vol/vol) in 500 μl of medium per well with 5 μM CpG 2216 for 24 hours. Data are shown as mean ± SEM of three independent experiments. Statistical significance was determined using paired Student's *t *test (*P < 0.05 **P < 0.01). **(B) **Human healthy PBMCs were preincubated for 15 minutes with BAY11 (10^-9 ^to 10^-7 ^M) or vehicle in the serum-free RPMI, and the serum of a SLE patient (at a final concentration of 20% vol/vol) with or without necrotic cell supernatant (at a final concentration of 20% vol/vol) was then added. After 24 hours, the concentrations of IFN-α in the culture supernatants were measured by ELISA. Data are shown as mean ± SEM of four independent donors. Statistical significance was determined using Mann-Whitney test (*P < 0.05 **P < 0.01).

Formation of immune complexes in serum consisting of autoantibodies and self-DNA in SLE continuously triggers the type I IFN production by blood pDCs, causing the development of the autoimmune process. Thus, the pDCs and serum represent the pathogenic cellular and humoral factors in SLE. In some previous *in vitro *experiments, stimulation of PBMCs with serum obtained from a patient with SLE induced IFN-α production, and serum containing DNA from necrotic cell supernatant enhanced the IFN-α production [35,36]. Based on these findings, we designed additional experiments using PBMCs and serum from patients with SLE, as described [36,37], to assess whether BAY11 functions as an inhibitor of type I IFN production under the pathophysiological condition of SLE. Initially, PBMCs from SLE patients were stimulated with CpG in the medium containing 20% auto-serum. Because blood pDCs in SLE are continuously triggered by serum immune complexes, the numbers of circulating pDCs are decreased and their function is defective [38,39]. Despite the low IFN response to CpG in lupus-PBMCs, we found that BAY11 had the ability to inhibit the IFN-α production even in the pathogenic PBMCs in a dose-dependent way (Figure 6A). Also in this experimental setting, 10^-5 ^M of BAY11 slightly induced PI-positive cells, but 10^-9 ^M to 10^-6 ^M of BAY11 did not increase PI expression in the PBMCs (data not shown). Next, we cultured healthy PBMCs with medium containing 20% of serum from SLE patients with or without 20% necrotic cell supernatants in the presence or absence of BAY11, and then measured the concentration of IFN-α. We preliminarily tested sera from three patients with active SLE having anti-double-stranded DNA antibody, and selected the best serum for inducing IFN-α by healthy PBMCs (data not shown). BAY11 inhibited the SLE serum-induced IFN-α production by PBMCs (Figure 6B). We next confirmed the observation that necrotic cell supernatants enhanced the SLE serum-induced IFN-α production by PBMCs (Figure 6B). BAY11 exerted the inhibitory function on the necrotic cell supernatant-enhanced IFN-α production from PBMCs in a dose-dependent way. We observed a similar inhibitory effect of BAY11 in this experimental setting using serum from two other SLE patients (data not shown). These data suggest that BAY11 has an inhibitory potential in relation to the pathogenic conditioned IFN-α production under *in vitro *experiments.

### BAY11 inhibits inducible IFN-α production *in vivo*

**Figure 7:**
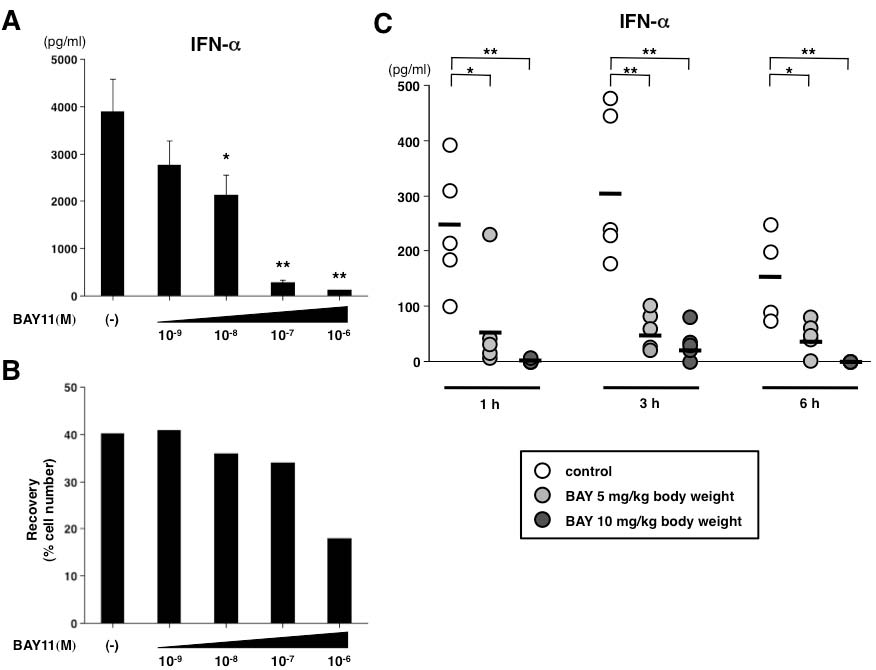
**BAY11 inhibits mouse pDC-derived IFN-α production and serum IFN-α elevation *in vivo***. **(A, B) **Sorted mouse splenic pDCs were preincubated for one hour with different concentrations of BAY11 (10^-9 ^to 10^-6 ^M) or vehicle. The pDCs were then cultured for a further 24 hours with poly U in complex with lipofectamine. (A) The concentrations of IFN-α in the culture supernatants were measured by ELISA. Data are shown as mean ± SEM of five independent experiments. (B) Viable cells were measured by a trypan-blue exclusion test. Similar results were observed in three independent experiments and the results of a representative experiment are shown. **(C) **C57BL/6 mice pretreated intraperitonealy with BAY11 (10 mg/kg or 5 mg/kg bodyweight) or vehicle for one hour, followed by intravenous injection of 50 μg/head poly U. Serum IFN-α was measured at several time points (one, three, and six hours). Bars indicate means of five independent experiments (except at six hours: n = 4). Statistical significance was determined using Mann-Whitney test (*P < 0.05 **P < 0.01).

Finally, to weigh up the possibility of inhibiting type I IFN production through BAY11 therapy in SLE, we evaluated the *in vivo *effect of BAY11 on the IFN response in mice. Preliminarily, we tested whether BAY11 functions in relation to mouse pDCs in the same way as in humans *in vitro*. We found that the production of IFN-α from sorted splenic pDCs of C57BL/6 mice in response to poly U in complex with lipofectamine was significantly decreased by the addition of BAY11 (Figure 7A). There was no difference in the rate of viable cells up to 10^-7 ^M of BAY11 (Figure 7B). Based on these *in vitro *findings, we next analyzed the serum IFN-α level at several time points after the injection of poly U in C57BL/6 mice pretreated with or without BAY11 (Figure 7C). Injecting mice with poly U rapidly increased the serum IFN-α level from one hour and continued to six hours after poly U injection. Pretreatment with both 5 mg/kg and 10 mg/kg of BAY11 prevented any serum IFN-α increases at all time points (one, three, and six hours). These data suggest that treatment with BAY11 could inhibit the *in vivo *IFN response by limiting pDC function when stimulated by TLR ligand.

## Discussion

The present study shows that IKK-neutralizing compound BAY11 affects IFN-α production mainly through its action on pDCs. IFN-α production is differentially regulated from other inflammatory cytokine production by the specific intracellular signaling under TLR activation [40]. A key molecular switch responsible for IFN-α synthesis in pDCs is the nuclear translocation of IRF7 [5]. We here found that BAY11 inhibits the nuclear translocation of IRF7 in pDCs and their IFN-α production. Although there are a number of reports showing the potential use of BAY11 in the treatment of malignancies through its inhibitory activity of NF-κB, the evidence linking it to autoimmune diseases is scant and there is no direct evidence so far that BAY11 prevents the activity of type I IFN-related diseases such as SLE. pDC activation in the blood by self-nucleic acids is regarded as a pathogenic trigger of the autoimmune process, and a dysregulated type I IFN elevation in serum by the continuous pDCs activation amplifies the pathogenic spiral in SLE [12-14]. On the basis of our current results showing that BAY11 inhibited the IFN-α production in PBMCs from SLE patients as well as from healthy donors, treatment with BAY11 may have the potential to attenuate the IFN environment and in turn to break off the pathogenic spiral in autoimmune diseases by limiting the disordered pDC function. Also, the experiments in injecting mice with poly U are suggestive of the agent's potential in inhibiting the inducible IFN response *in vivo*, though the serum IFN elevation is not pathophysiologically but artificially induced in our experimental setting.

Under normal physiological conditions, host-derived self-nucleic acids usually have little chance of encountering endosomal TLR7 and TLR9 because of their instability in relation to nucleases and by their location separate from endosomes. However, a breakdown in the innate tolerance to self-nucleic acids occurs when tissue injury or necrosis release some endogenous molecules, including antimicrobial peptide (LL37) and nuclear protein (high-mobility group box 1 protein; HMGB1), which help to promote stabilization and delivery of immune complexes into early endosomes [9,41,42]. Even in the current experiments using SLE sera and necrotic cell supernatant that perhaps comprise these molecules, BAY11 functions as an inhibitor of the pathogenic IFN-α response. Thus, our findings provide an opportunity for the development of therapeutic strategies that directly inhibit the pathogenic cellular and molecular components leading to SLE.

Also TNF-α production in pDCs was repressed by BAY11 at the high concentration, and accordingly the therapeutic window of BAY11 for selective interference with IFN-α was narrow. Since endogenous TNF-α limits the IFN-α production in pDCs [43], there is a possibility that the repression of TNF-α results in abating the inhibitory function of BAY11 against IFN-α production at high concentration. Thus, the most efficient and practical biological concentration may need to be decided from further studies.

At the downstream of TLR7/9-MyD88, the signaling pathway bifurcates into NF-κB- and IRF-7- activation pathways, which are responsible for the induction of proinflammatory cytokines and type I IFNs, respectively [2,5]. Whereas IRF7 phosphorylation and nuclear translocation depend on IKKα, NF-κB activation needs IKKβ. IKKβ homodimer can compensate the function of heterodimer of IKKα and IKKβ in activating NF-κB in the absence of IKKα [40]. Given the function of BAY11 as an inhibitor of IKK activity [18,25], a more plausible explanation for its inhibitory activities in regards to both IFN-α and TNF-α in pDCs is that BAY11 targets IKKα in the inhibition of IFN-α and IKKβ in the inhibition of TNF-α at the downstream of TLR7/9-MyD88.

The other two IKK-related kinases, TANK-binding kinase 1 (TBK1) and IKKτ (also called as IKKε), are also reported to be involved in the phosphorylation of IRF-7 as well as IRF3 [44]. However, CpG-induced IFN-α secretion is not impaired in mice deficient in TBK1 or IKKτ [7], indicating that these two IKKs are dispensable for TLR-mediated induction of IFN-α in pDCs. Similar to IKKα deficiency, IRAK1 deficiency leads to the defective transcriptional activation of IRF7 and defective production of IFN-α gene in pDCs [8], indicating a critical involvement of IRAK1 in the induction of type I IFNs in TLR7 and TLR9 signaling pathways. Although it is unclear at present how IKKα links to IRAK1, either kinase appears to be the gateway for activation of IRF7 to induce IFN-α production in pDCs and both could be potential targets for the treatment of autoimmune disorders. Further studies will be required to determine what the specific target of BAY11 is, whether BAY11 inhibits IRAK1 activationor the precise mechanism by which BAY11 inhibits the signaling pathway of TLR-mediated IFN-α production in pDCs.

In contrast to RNA-sensing receptor TLR7 in pDCs, another RNA-sensing cytosolic RIG-I-like receptor sensors in myeloid DCs through recognition of dsRNA such as poly IC can also induce IFN-α/β in an IPS-1-dependent manner [45]. However, BAY11 was incapable of inhibiting the poly IC-induced IFN-α production by myeloid DCs. This finding can be explained by the evidence that, at the downstream of RIG/MDA5-IPS-1, both IKKα and IKKβ are dispensable for the type I IFN production [9,32]. However, BAY11 could suppress the poly IC-induced IL-12 and TNF-α secretion by the myeloid DCs. This could also be explained by the evidence showing that TLR3-mediated production of proinflammatory cytokines is dependent on IKKβ during the signaling process of the TRIF-NF-κB pathway [40].

## Conclusions

Collectively, our data demonstrated an antagonistic property of BAY11 to the *in vitro *and *in vivo *IFN response and imply a possibility for new therapeutic approaches by interference with the pathogenic components of autoimmune disorders. Thus, our findings provide a foundation for the exploitation of novel IFN inhibitors, and we here propose that a selective IKKα inhibitor designed to abrogate nuclear translocation of IRF7 and sequential type I IFN production would be a promising tool for the treatment of IFN-related diseases.

## Abbreviations

BAY11: BAY11-7082; BDCA: blood dendritic cell antigen; DC: dendritic cells; IFN: interferon; IKK: IκB kinase; IRF7: IFN regulatory factor 7; PBMC: peripheral blood mononuclear cells; pDC: plasmacytoid dendritic cell; SLE: systemic lupus erythematosus; TLR: toll-like receptor; TNF: tumor necrosis factor.

## Competing interests

The authors declare that they have no competing interests.

## Authors' contributions

RM performed the experiments and wrote the paper. TI planned, designed and wrote the paper. SN, RA, TK, and SF contributed to the experimental planning and design. HA, YK, MO, NM, and KS performed the experiments of human cells. CY and KH performed the mouse experiments. All authors read and approved the final manuscript.
